# Wayward youth: how maturity, reproduction and seaweed drive snapper (*Lutjanus* spp.) habitat shifts

**DOI:** 10.1111/jfb.70212

**Published:** 2025-09-08

**Authors:** Laughlin Siceloff, Matthew S. Kendall, Clayton Pollock, Mark E. Monaco

**Affiliations:** ^1^ CSS Inc. Fairfax Virginia USA; ^2^ National Oceanic and Atmospheric Administration/NOS/NCCOS/MSE/Biogeography Branch Silver Spring Maryland USA; ^3^ National Park Service Christiansted Virgin Islands USA

**Keywords:** lutjanidae, spawning, mangroves, ontogeny, sargassum

## Abstract

Despite snappers' (family Lutjanidae) commercial and ecological significance, knowledge gaps remain regarding life history, ontogeny and ecology across their range in the Caribbean and south Atlantic. There is also a need to explore the efficacy of marine protected areas (MPAs) as a tool for enhancing nursery and spawning habitat conservation for multiple snapper species. Additionally, even as hurricanes and sargassum inundation have become rising issues for coastal communities, there is a scarcity of data on how commercially important species respond to these environmental disturbances. To address these data gaps, we investigated the spatial and temporal movements of 32 snappers of multiple species in mangrove estuary, reef and shelf edge habitats in St Croix, US Virgin Islands for up to a year using surgically implanted acoustic transmitters and hydrophone arrays. We documented ontogenetic habitat shifts as individuals moved incrementally from juvenile mangrove habitat to adult reef habitat, and several were tracked migrating >30 km to a potential spawning site. Results demonstrated the connectivity of a series of MPAs and their management potential across lutjanid life stages. Size and growth estimates during these movements highlighted the regional variability in lutjanid ontogeny and the need for population‐specific life‐history studies. Snapper displayed no change in behaviour during a direct hurricane impact, but a significant number of fish made temporary or permanent habitat shifts coinciding with a severe sargassum event inside a bay, providing one of the first descriptions of fishes' behavioural responses to coastal sargassum inundation.

## 1 INTRODUCTION

Although snappers (family Lutjanidae) are commonly occurring fish species on reef ecosystems in the Caribbean and Atlantic, many gaps remain in our knowledge of important aspects of their life history. In general, studies tend to be compartmentalized within life stages and focus either on the juvenile life stages, which often occur in mangrove and seagrass habitat (Adams & Tobias, 1999; Faunce & Serafy, 2007; Nagelkerken et al., 2000; Nagelkerken et al., 2002), or on adult life stages that occur on reefs with an emphasis on spawning (Bacheler et al., 2020; Feeley et al., 2018; França et al., 2021). Seldom are the transitions between life stages examined, such as the ontogenetic habitat shifts from late juvenile to adult phases (Huijbers et al., 2015) or the movements back and forth between adult resident areas and spawning habitats (Biggs & Nemeth, 2016; Feeley et al., 2018; Heidmann et al., 2024; Luo et al., 2009). Little is known about species‐specific habitat preferences and movement in response to environmental stressors like severe storms and algal blooms, even as these events may intensify with climate change (Gobler, 2020; Villarini & Vecchi, 2012; Wang & Wu, 2013). Furthermore, what is known about ontogeny, life history and ecology for snapper species comes from a limited number of populations and study sites, leaving spatial data gaps across their ranges.

Snapper habitat preferences vary across ontogeny, yet habitat shifts are rarely observed and poorly understood. Instead, these shifts are inferred from surveys that find size classes spatially separated by habitat type (Bacheler et al., 2020; Cocheret de la Morinière et al., 2002; Flaherty‐Walia et al., 2015), or isotope and diet analyses that link life stages to different habitats (Cocheret de la Morinière, Pollux, Nagelkerken, Hemminga, et al., 2003; Mateo et al., 2010; Verweij et al., 2008). Snapper may utilize multiple habitats in their home range (Huijbers et al., 2015; Verweij et al., 2007) as seen in diel movements between mangrove or reef shelter by day and seagrass foraging grounds at night (Luo et al., 2009; Nagelkerken et al., 2000), yet permanent habitat shifts are rarely documented in movement studies. Furthermore, habitat utilization is often known from only a few study sites despite the fact that these trends may vary by location and population. Dog snapper *Lutjanus jocu* (Bloch & Schneider 1801) is one species that exemplifies such data gaps. Knowledge of their ecology comes mostly from Brazilian populations (Menezes et al., 2022) where juveniles are found in mangrove nursery habitat and adults are found on progressively deeper reefs with age (Moura et al., 2011; Pimentel & Joyeux, 2010). Their habitat preferences with ontogeny are poorly studied in the northern hemisphere, however, and their movement patterns are largely unknown in any region. Long‐term movement tracking (e.g. passive acoustic telemetry) can chronicle when and how lutjanids including *L. jocu* move to new habitats as they mature, and the behaviours, habitat preferences and distances associated with these shifts.

Like ontogeny, reproduction can drive movement to new locations, yet life history and reproductive ecology can vary widely and have only been described for select populations despite their importance for conservation and management. Size at maturity for a given species may vary dramatically by study and location (Martinez‐Andrade, 2003). Currently, no size at maturity assessments exist for US Caribbean *Lutjanus* spp. populations. A recent estimation of snapper life‐history parameters for these islands relies on Florida data for many species and relies on Brazilian and Cuban studies for *L. jocu* (Stevens et al., 2019). Spawning timing varies for species like *L. jocu*, whose spawning can peak in winter (Kadison et al., 2006), spring (Heyman & Kjerfve, 2008) or summer (Claro & Lindeman, 2003; França et al., 2021; Lindeman et al., 2000) depending on the location. With few exceptions (Biggs & Nemeth, 2016; Feeley et al., 2018; Pittman et al., 2014), connectivity between spawning aggregation sites and year‐round habitat, and where aggregating fishes arrive from or disperse to, is poorly understood throughout the western Atlantic. Other than studies of the Grammanik Bank spawning grounds off of St Thomas (Biggs & Nemeth, 2016; Kadison et al., 2006) and the mutton snapper *Lutjanus analis* (Cuvier 1828) spawning aggregation site on the southwest shelf of St Croix (Heidmann et al., 2024), the US Caribbean represents an important spatial gap in reproductive knowledge for lutjanids. As shown by *L. jocu* spawning times across its range, regional variability ensures reproductive information cannot be accurately inferred from other places and must be addressed through local data collection.

Apart from ontogenetic preferences, spawning and foraging, other factors such as environmental disturbances can influence fish movement between habitats. Hurricanes are well‐known disturbances that drive fish species to other locations (Bacheler et al., 2020; Patterson III et al., 2001; Udyawer et al., 2013). Floating sargassum blooms and resulting ‘brown tide’ coastal incursions are another environmental disturbance occurring with increasing frequency and magnitude across the Caribbean since 2011 (Franks et al., 2016; Hu et al., 2016; Wang et al., 2019). Sargassum inundation on coastlines is known to negatively impact coastal economies (Bartlett & Elmer, 2021; Robledo et al., 2021), harm coastal fisheries (Cox et al., 2019; Ramlogan et al., 2017), pose a health risk to humans (Resiere et al., 2019), transport toxic metals into coastal ecosystems (Rodriguez‐Martinez et al., 2020), cause coral and seagrass mortality (van Tussenbroek et al., 2017) and kill various other marine organisms from eutrophication and hypoxia caused by decomposition, including in the US Virgin Islands (Cruz‐Rivera et al., 2015; Rodriguez‐Martinez et al., 2019). Yet to date, there is little information about how fishes in coastal areas respond to sargassum events and how their response might impact local fisheries in both the short and long term.

For these and other reasons, fishes move across habitats and change location over their lifespans. Marine protected areas (MPAs) can provide conservation benefits to fish populations on nursery grounds (Heupel & Simpfendorfer, 2005), adult reef habitat (McCook et al., 2010) or spawning aggregation sites (Erisman et al., 2015), but a single MPA may not encompass all of these habitats. Ontogenetic migrations can limit MPA efficacy (Grüss et al., 2011) but a well‐designed network of MPAs across different habitats may offer effective protection at different life‐history stages, as long as the linkages between habitats are well understood (Grüss et al., 2011; Roberts et al., 2003; White, 2015). With sufficient spatial and temporal coverage in its study design, an acoustic telemetry study can be an effective tool to address many of these issues and document ontogenetic migrations between MPAs, evaluate connectivity across an MPA network (Espinoza et al., 2015; Pittman et al., 2014), and evaluate the timing and placement of seasonal protections (Heidmann et al., 2024).

Our study used passive acoustic telemetry to fill several regional information gaps on spatial and temporal movement patterns of multiple snapper species during the late‐juvenile to early‐adult life stages. We investigated movements within a mangrove‐lined estuary, to an adjacent reef and along the coast to potential spawning habitat. We sought to understand temporal patterns of behaviour and habitat preference (diel and seasonal) and to chronicle ontogenetic changes to habitat preference and location over time. Furthermore, we describe possible migration pathways and spawning grounds for snapper and fish size at the time of potential spawning movements and provide evidence of size at maturity for St Croix populations. We also document the fine‐scale behavioural responses to two unexpected disturbances: the impact of a hurricane and a notable sargassum inundation and decomposition event. We describe the movements and connectivity between a series of MPAs for commercially important species and the potential for this US Caribbean MPA corridor to confer protection at different life stages. Finally, we related all of these findings to those in other locations to place US Caribbean lutjanids in a greater regional context and provide a more complete understanding of lutjanid ecology across their western Atlantic/Caribbean range.

## 2 MATERIALS AND METHODS

### 2.1 Ethical statement

Animal collection, care and research complied with National Oceanic and Atmospheric Administration (NOAA) National Environmental Policy Act protocols and animal welfare guidelines as policies as approved by the National Park Service (SARI‐2018‐SCI‐0001 & SER_SARI_Kendall_Fish_2018) and the US Virgin Islands Department of Planning and Natural Resources (CZM17040X).

### 2.2 Study area

**FIGURE 1:**
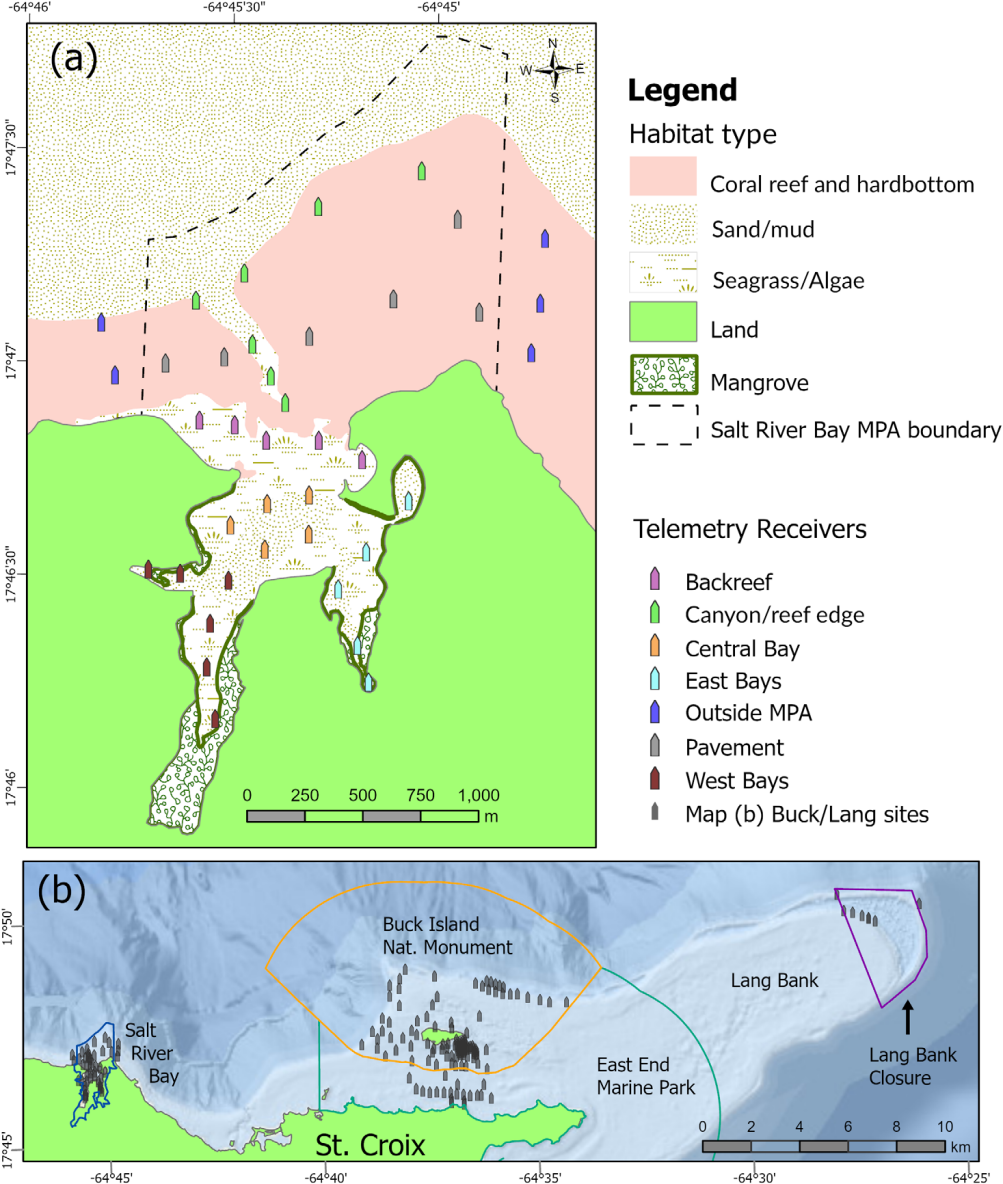
(a) Habitats and acoustic receiver locations within the Salt River Bay National Historical Park and Ecological Preserve and (b) the positions of acoustic receivers around Buck Island and Lang Bank, and various marine protected area (MPA) boundaries.

This study of fish movements was conducted using two telemetry arrays deployed along the northeast coast of the Caribbean island of St Croix, US Virgin Islands. First, we installed a dense array of 37 acoustic receivers (model VR2W; InnovaSea Systems Inc.) in May 2017 within Salt River Bay National Historical Park and Ecological Preserve (NHEP), hereafter referred to as the Salt River array (Figure 1a). The preserve was established in 1992 by the National Park Service, in part to preserve the bay's biological resources (United States Congress, 1992). Salt River Bay is lined with one of the last and largest mangrove habitats in St Croix (Kendall et al., 2005) and is a known reef fish nursery (Adams & Tobias, 1999; Kendall, Williams, Ruffo, et al., 2021). Salt River Bay is composed of several smaller bays around a central basin that are all 2–5 m deep, have a mud or sand bottom, seagrass or sparse algae and are lined with red mangroves *Rhizophora mangle* (Kendall et al., 2005). Seagrass and occasional hardbottom on the backreef transition to hardbottom reef habitat outside of the bay, characterized by colonized pavement typically <25 m deep extending north to a rugose, aggregate reef shelf edge. An underwater canyon 25–300 m deep with steep, rugose walls runs from a channel in the backreef out to the shelf edge, connecting the bay to the open ocean (Kendall et al., 2005).

We strategically placed passive acoustic receivers throughout the system to monitor fish locations within this MPA and on pavement east and west of its boundaries (*n* = 21 inside the bay, *n* = 18 outside) (Kendall, Siceloff, Monaco, et al., 2021; Kendall, Siceloff, Ruffo, et al., 2021) (Figure 1a). Receivers were spaced approximately evenly (200–250 m apart) in a tiered arrangement from the bays, backreef and offshore to detect fish passage. Detection range tests (Kendall, Siceloff, Monaco, et al., 2021) revealed the 50% probability of detection distance to be ~175 m inside the bay, indicating overlapping receiver coverage and effective monitoring of most bay areas. On the reef and pavement outside the bay, the range at 50% detection probability was ~100 m, leaving gaps in coverage between many adjacent receivers. A sentinel tag deployed in the central bay was detected every day in 2018 and there was no difference in day versus night detections. This made accounting for seasonal or diel bias during analysis unnecessary (Kendall, Siceloff, Monaco, et al., 2021). We retrieved cleaned, and downloaded receivers every 6 months and removed the array on 24 June 2019 (a 2‐year study period).

The second array we utilized was composed of >100 receivers from the collaborative US Caribbean Acoustic Network (USCAN). These were deployed intermittently along the northeastern coast of St Croix, including locations around Buck Island, Buck Island Channel and eastward to the tip of Lang Bank (hereafter referred to as the Buck/Lang array) (Figure 1b). This region consists of a mix of coral reefs, flat hardbottom, sand and seagrass in waters typically <25 m deep. Much of this array was within the Buck Island Reef National Monument, a no‐take MPA, and several receivers were within the eastern Lang Bank closure designed to protect red hind *Epinephelus guttatus* (L. 1758) spawning, which is no‐take from December 1 to February 28 and has a year‐round ban on bottom‐fishing gear (Federal Register, 1993) (Figure 1b). Between these two closures lies the St Croix East End Marine Park (EEMP) enclosing the eastern half of St Croix (Legislature of the Virgin Islands, 2002) (Figure 1b). The EEMP includes a no‐take area encircling its shoreline, but it extends <1 km from shore and comprises a small portion of the park (Brown et al., 2016; The Nature Conservancy, 2002). The various institutions in USCAN managed and maintained their own receivers for specific research objectives, and therefore some Buck/Lang array locations were not in place consistently for the duration of our study. All USCAN receivers on the north coast were east of Salt River Bay, and thus there was no receiver coverage west of the study area. Despite the irregular timing and distribution of Buck/Lang receivers, they provided an important opportunity to detect potential fish movements between the high‐density Salt River array and a known spawning area for reef species, including *E. guttatus* and queen triggerfish *Balistes vetula* L. 1758, on the eastern side of Lang Bank (Bryan et al., 2019; Nemeth et al., 2006).

### 2.3 Fish tagging and maturity estimation

All lutjanid species that we tracked in this study were captured inside Salt River Bay NHEP using hook and line or fish traps between May 2017 and July 2018. These included *Lutjanus jocu*, *Lutjanus analis*, schoolmaster snapper *Lutjanus apodus* (Walbaum 1792), grey snapper *Lutjanus griseus* (L. 1758) and lane snapper *Lutjanus synagris* (L. 1758). We surgically implanted coded acoustic transmitters (InnovaSea model V8‐4L, random ping delay 130–230 s, ~324‐day battery life) into the body cavity of subject fish through a 1.5‐cm ventral incision closed with a surgeon's knot. After the 1–2 min procedure, we moved tagged individuals to a shaded recovery bin until normal respiratory and swimming behaviours resumed, then released each fish at the point of capture.

At the time of capture we measured total length (TL) and categorized each fish as a juvenile or potential adult based on best available size at maturity values. Given that so few life‐history assessments have been conducted on US Caribbean reef fish populations, these values were obtained from studies conducted in other regions, usually Florida and Gulf of Mexico population data but also Brazil. Where necessary, we converted fork length to total length using length–length conversions for *L. jocu* (Fröse & Pauly, 2024; Martinez‐Andrade, 2003), *L. griseus* (SEDAR, 2022), *L. apodus* (Fröse & Pauly, 2024; Martinez‐Andrade, 2003) and *L. synagris* (Fröse & Pauly, 2024; Martinez‐Andrade, 2003). Using best available values, *L. jocu* total length at 50% maturity (*L*_m_) was estimated at 50.35 cm TL (Stevens et al., 2019) with a minimum size at maturity of 32 cm (Freitas et al., 2011). *Lutjanus griseus L*_m_ was 28.3 cm (SEDAR, 2022) with a 24.1 cm minimum (Macal‐López et al., 2022), *L. synagris L*_m_ was 26.1 cm (Stevens et al., 2019) with an 18.3 cm minimum (Freitas et al., 2014), *L. analis L*_m_ was 42.2 cm with a 40.5 cm minimum (SEDAR, 2024), and *L. apodus L*_m_ was 25.5 cm (no minimum available; Stevens et al., 2019). We assumed a fish to be potentially mature if equal to or above the minimum reported size at maturity, or *L*_m_ if a minimum value was not available. We estimated age at the time of tagging using Von Bertalanffy growth equations for each species (Manooch & Mason, 1984; Potts et al., 2016; Potts & Burton, 2017; SEDAR, 2022, 2024). We also used these growth equations and maturity values to estimate length and infer maturity status when individuals made habitat shifts or emigrated from the study area.

### 2.4 Analysis

We formatted and organized raw detection data through the FACT Network's research node workspace (Young et al., 2020) and the GLATOS package in R (Krueger et al., 2018), and analysed data using the statistical software R 4.3.0 (R Core Team, 2023) and Esri ArcGIS Pro 3.3. We visualized daily and seasonal movement patterns for each fish with customized abacus plots and grouped receivers by location into subarrays to simplify plots and reflect the inshore to offshore locations of individuals (Figure 1a). Each subarray consisted of five or six regularly spaced receivers except for the Buck/Lang array, which covered a much larger area and was more irregularly spaced. Detection days (i.e. each date with one or more detections) for each fish were coded by receiver group and whether the detection occurred during the daytime, nighttime or both using local sunrise and sunset times.

#### 2.4.1 Movement types

We summarized and analysed diel fish movements by first determining whether individual fish were detected on a different receiver subarray during the day or night. For this step, individuals could be detected exclusively on different receivers during the day or night, only detected during the day with nighttime locations being unknown or vice versa. To determine if fish were more active during the night than the day, such as for nocturnal foraging across seagrass habitat, we applied a paired *t*‐test with a Shapiro–Wilk test for normality to evaluate the difference in the mean number of receivers with detections at night versus the day for each species.

Next, we identified the dates of ontogenetic shifts, which we defined as any change in location or habitat that persisted for a month or more. We also noted the location of each ontogenetic shift and sorted it into a movement direction based on the initial location, e.g. from bay (nursery habitat) to canyon/reef (adult habitat) or vice versa. In addition, we observed another category of short‐term movement, characterized by an excursion to a new location lasting days to weeks before a return to the previous area.

We also noted movements out of the Salt River array. Fish were scored as ‘Yes’ (direct evidence with offshore movement across multiple Salt River array receivers before disappearing and/or detections observed on Buck/Lang receivers elsewhere around St. Croix), ‘Possible’ (detections ceased before battery expiration, but no direct evidence of leaving) or ‘No’ (fish detected in the Salt River Bay array regularly until expiration of transmitter battery). If a fish was detected on Buck/Lang receivers, we characterized that movement between arrays in four additional ways. These included the direction of movement (i.e. eastward vs. westward), the lunar phase on the date when movement began and the speed of movement calculated using the straight‐line distance and time between detections on consecutive receivers. We also classified the time of day that movement began based on local sunrise and sunset times as ‘day’ or ‘night/twilight’ (including 2 h before sunset and 2 h after sunrise).

#### 2.4.2 Weekly movements

To determine if the weekly timing of fish movements was coordinated or synchronized and was significantly different from random, we used a resampling permutation test (Good, 2005). This simulation test determined the probability that the observed number of fish movements during any particular week of the tracking period could have occurred by random chance. There were not enough fish tagged in 2017 to analyse the first year of the study, but 25 of the fish tracked in sampling year 2018–2019 had enough detectable movements to enable this analysis during that year. A total of 38 distinct movements from these 25 individuals were tallied into weekly bins for the entire tracking span from the date the first fish was tagged in April 2018 through March 2019 when most tags expired. The number of fish movements in each week was totalled to represent the observed pattern of fish movements. For example, all 25 fish were actively being tracked during the week beginning 28 July 2018 and only one of them made some type of movement. This observed weekly pattern was tested against a random distribution of the same number of fish movements in the same tracking span. For this simulation, the observed total number of 38 fish movements in the tracking period was randomly assigned to the weekly tracking bins and the results were tallied. This randomization of fish movements was repeated and tallied 100 times. This enabled us to determine the number of times that the observed pattern of weekly fish movements could have arisen by chance. The result was expressed as a *p* value (*α* = 0.05) which indicated the percentage of the 100 random movement simulations that met or exceeded the observed proportion of movements for that week. For example, in the week beginning 28 July, 63% (*p* = 0.63) of the simulations had one or more movements. This indicates that the observed pattern could have happened at random very often and is interpreted as a non‐significant result. When less than 5% (*p* < 0.05) of the simulations exceeded the observed pattern, that week was considered to have had significantly more fish movements than could have been expected to occur if fish were relocating randomly.

#### 2.4.3 Environmental disturbances

To gather more information about environmental phenomena that potentially correlated with fish movements, we examined local daily records and tropical storm tracking data from the National Weather Service and monthly remote sensing maps from the Coastal Ocean Observing System (CARICOOS) for significant rainfall events or changes in the chlorophyll index or wind speed. In addition, we contacted full‐time residents of St Croix who were present throughout the duration of our study and asked them for any relevant information on environmental disturbances in the study area. This informal survey focused on two groups of individuals: National Park Service employees responsible for managing the Salt River Bay NHEP, and staff and management of the Salt River Bay Marina. We provided them with movement dates and requested that they look back at their records for events including marina construction, derelict boat removal or other unusual events.

## 3 RESULTS

Thirty‐two lutjanids were tagged, detected regularly for >1 month and included in analyses. This consisted of 14 *L. jocu*, 10 *L. apodus*, five *L. griseus*, two *L. synagris* and one *L. analis*. Total length at tagging ranged from 20 to 39 cm TL, and all potentially mature sizes are noted in Table 1. Sixteen fish (50%) were immature and primarily caught in mangrove habitat inside the bay, including the majority of *L. jocu* (*n* = 11). The remainder were potentially mature (TL > minimum size at maturity) and were evenly split between the bay and the reef.

**TABLE 1:** Tagging information and types of movements observed for all snapper analysed in this study, including *Lutjanus analis* (LAN), *Lutjanus apodus* (LAP), *Lutjanus griseus* (LGR), *Lutjanus jocu* (LJO) and *Lutjanus synagris* (LSY).

Fish ID	Snapper species	Location tagged	TL at tagging	Date tagged	Diel pattern	Excursion	Ontogenetic shift	Emigration	TL at emigration	Detection days
LAN 31	*L. analis*	Backreef	30	04/24/18	Y	N	Y	P	33	120
LAP 04	*L. apodus*	Pavement	30^†^	04/26/18	Y	N	N	N	n/a	219
LAP 05	*L. apodus*	Pavement	27^†^	04/26/18	Y	N	N	N	n/a	131
**LAP 07**	*L. apodus*	Pavement	28^†^	04/26/18	Y	N	Y	Y	29^†^	95
LAP 28	*L. apodus*	Pavement	27^†^	04/26/18	Y	N	N	N	n/a	107
LAP 38	*L. apodus*	Central Bay	25^†^	05/17/18	N	N	N	N	n/a	200
LAP 40	*L. apodus*	Pavement	27^†^	05/17/18	Y	N	P	P	29^†^	28
LAP 77	*L. apodus*	Backreef	28^†^	04/25/18	Y	N	N	N	n/a	324
LAP 422	*L. apodus*	Central Bay	20	04/25/17	N	N	P	P	n/a	63
LAP 457	*L. apodus*	Pavement	22	05/24/17	Y	N	P	N	n/a	34
LAP 466	*L. apodus*	Central Bay	21	05/23/17	N	N	P	P	22	27
LGR 41	*L. griseus*	East Bays	20	07/26/18	Y	N	Y	N	n/a	322
**LGR 48**	*L. griseus*	West Bays	27^†^	05/16/18	Y	N	Y	Y	28^†^	116
LGR 75	*L. griseus*	Canyon	29^†^	07/26/18	N	N	P	P	30^†^	37
LGR 440	*L. griseus*	Central Bay	34^†^	04/26/17	Y	N	N	N	n/a	338
LGR 441	*L. griseus*	Central Bay	33^†^	04/26/17	Y	N	N	N	n/a	351
LJO 06	*L. jocu*	West Bays	20	04/24/18	Y	N	Y	Y	24	145
LJO 08	*L. jocu*	West Bays	22	04/24/18	N	N	Y	Y	29	189
LJO 09	*L. jocu*	West Bays	39^†^	04/24/18	Y	Y	Y	Y	43^†^	201
LJO 27	*L. jocu*	West Bays	39^†^	04/23/18	Y	N	Y	N	n/a	331
**LJO 32**	*L. jocu*	West Bays	31	07/26/18	N	Y	Y	Y	32^†^	110
LJO 33	*L. jocu*	West Bays	35^†^	04/23/18	Y	Y	N	N	n/a	330
**LJO 35**	*L. jocu*	West Bays	29	04/23/18	N	Y	Y	Y	34^†^	208
**LJO 50**	*L. jocu*	East Bays	28	04/20/18	N	Y	N	P	33^†^	204
LJO 53	*L. jocu*	West Bays	23	04/24/18	N	N	Y	Y	28	169
**LJO 55**	*L. jocu*	West Bays	26	04/24/18	N	Y	Y	Y	29	298
LJO 56	*L. jocu*	West Bays	20	04/25/18	N	N	N	N	n/a	336
LJO 68	*L. jocu*	West Bays	26	04/21/18	Y	N	Y	N	n/a	335
LJO 69	*L. jocu*	West Bays	27	04/21/18	N	Y	Y	Y	30	129
LJO 73	*L. jocu*	West Bays	25	04/23/18	N	Y	N	N	n/a	331
LSY 11	*L. synagris*	Canyon	27^†^	04/18/18	N	N	Y	N	n/a	331
LSY 423	*L. synagris*	West Bays	23^†^	04/24/17	N	Y	N	P	23^†^	42

*Note*: Fish IDs in bold indicate fish detected on the Buck/Lang array. Total length measured in cm. † indicates lengths greater than or equal to the minimum size at maturity reported for each species or the minimum *L*_m_ value in the case of *L. apodus*.

Abbreviations: P, possible movement; N, no; n/a, not applicable; TL, total length; Y, yes.

### 3.1 Diel activity patterns

**FIGURE 2:**
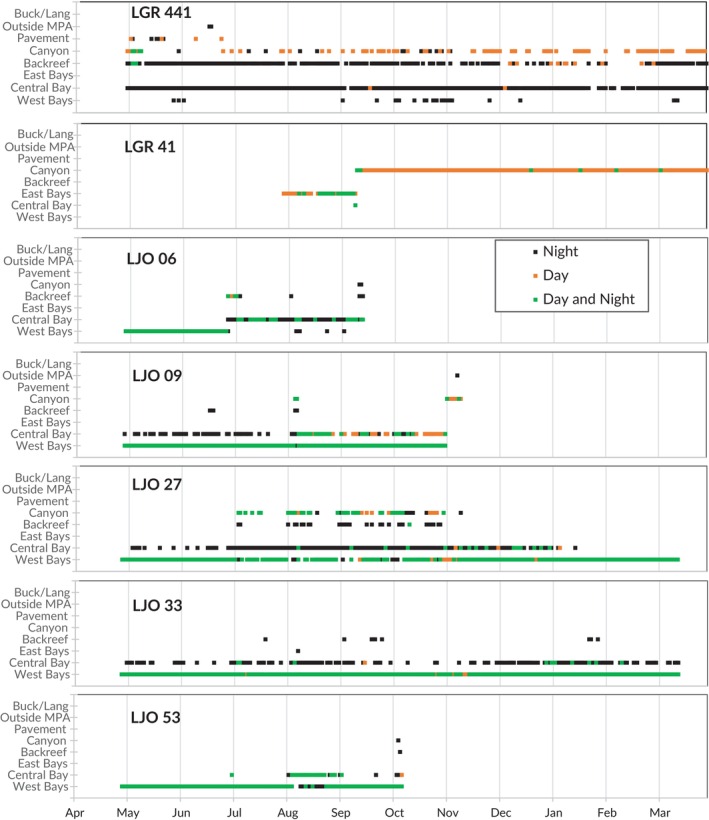
Daily detection plots for a subset of snapper showing examples of movement patterns and habitat shifts across the study area, including *Lutjanus apodus* (LAP), *Lutjanus griseus* (LGR) and *Lutjanus jocu* (LJO). LGR 441 track was from 2017 to 2018; all others were from 2018 to 2019. LGR 441 and LGR 41 detections continued beyond the time ranges shown here. MPA, marine protected area.

**FIGURE 3:**
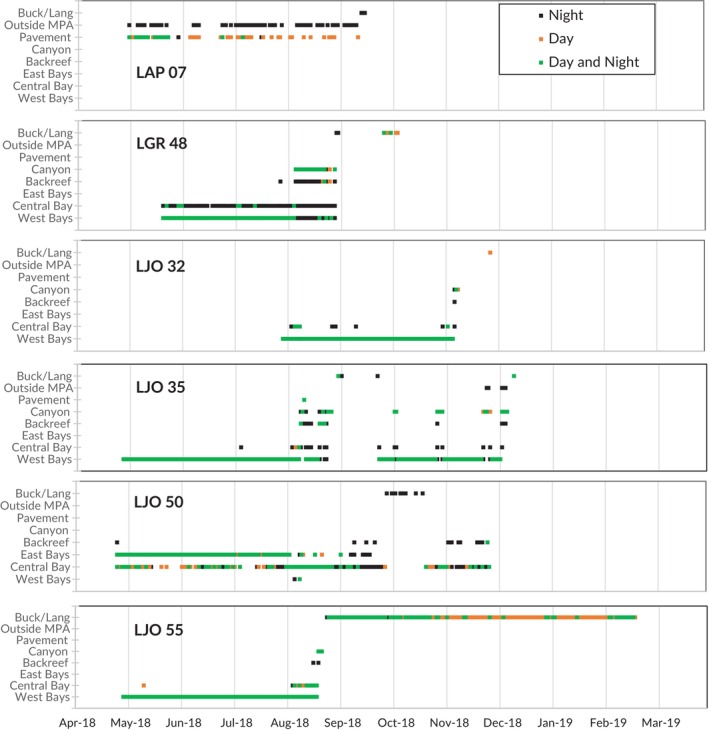
Daily detection plots for the six snapper tracked migrating outside of Salt River Bay along the Buck/Lang array, including *Lutjanus apodus* (LAP), *Lutjanus griseus* (LGR) and *Lutjanus jocu* (LJO). MPA, marine protected area.

Half (*n* = 17) of fish exhibited a clear diel difference in habitat use for ≥50% of their tracking span (Table 1) where different receivers or subarrays were used during the day versus night. Abacus plots of individual fish tracks (Figures 2 and 3) show representative examples of diel movement patterns and habitat shifts over time (all remaining abacus plots are included in Supporting Information Data [Link], [Link]). Five fish were resident to the western marina mangroves by day but active in the central bay by night (e.g. LJO 33; Figure 2), and four fish were resident to the reef and canyon structure by day but similarly active in the central bay and backreef by night (e.g. LGR 441; Figure 2). One fish on the reef pavement shifted inshore at night without entering the central bay, and another shifted between the central bay's mangrove edge by day and the bay's seagrass centre by night. Six fish tagged on the reef pavement were detected there either by day or by night, but not both, indicating diel changes in location for this group as well (e.g. LAP 05; Figure 2).

The average number of receivers that *L. jocu* were detected on during the day versus night was significantly different (paired *t* = 4.15, *df* = 13, *p* < 0.001) such that 12 of the 14 fish were detected on more receivers at night (mean 2.7 receivers) than during the day (mean 1.8 receivers), and these additional receivers reached at night tended to be in seagrass habitat. There was no significant difference in average number of day versus night receivers used by *L. apodus* (paired *t* = 0.9, *df* = 9, *p* < 0.39). No other species had sufficient sample size to perform this test.

### 3.2 Ontogenetic shifts

Fifteen fish (47% of the total), most of which were immature at the time of tagging, made one or more ontogenetic shifts (Table 1) wherein they moved from mangrove bay nursery habitat to more seaward habitats like the reef canyon (e.g. LGR 41, LJO 06, LJO 27; Figure 2). This included 71% of *L. jocu* but only 10% of *L. apodus*, the majority of which were resident to the reef and likely adults when tagged (Table 1). Two *L. jocu* made ‘reverse’ ontogenetic shifts where they gradually expanded their activity range from solely mangrove habitat to central bay and reef canyon for months, only to eventually contract that range back to a mangrove bay again (e.g. LJO 27; Figure 2).

### 3.3 Migrations and potential spawning activity

**FIGURE 4:**
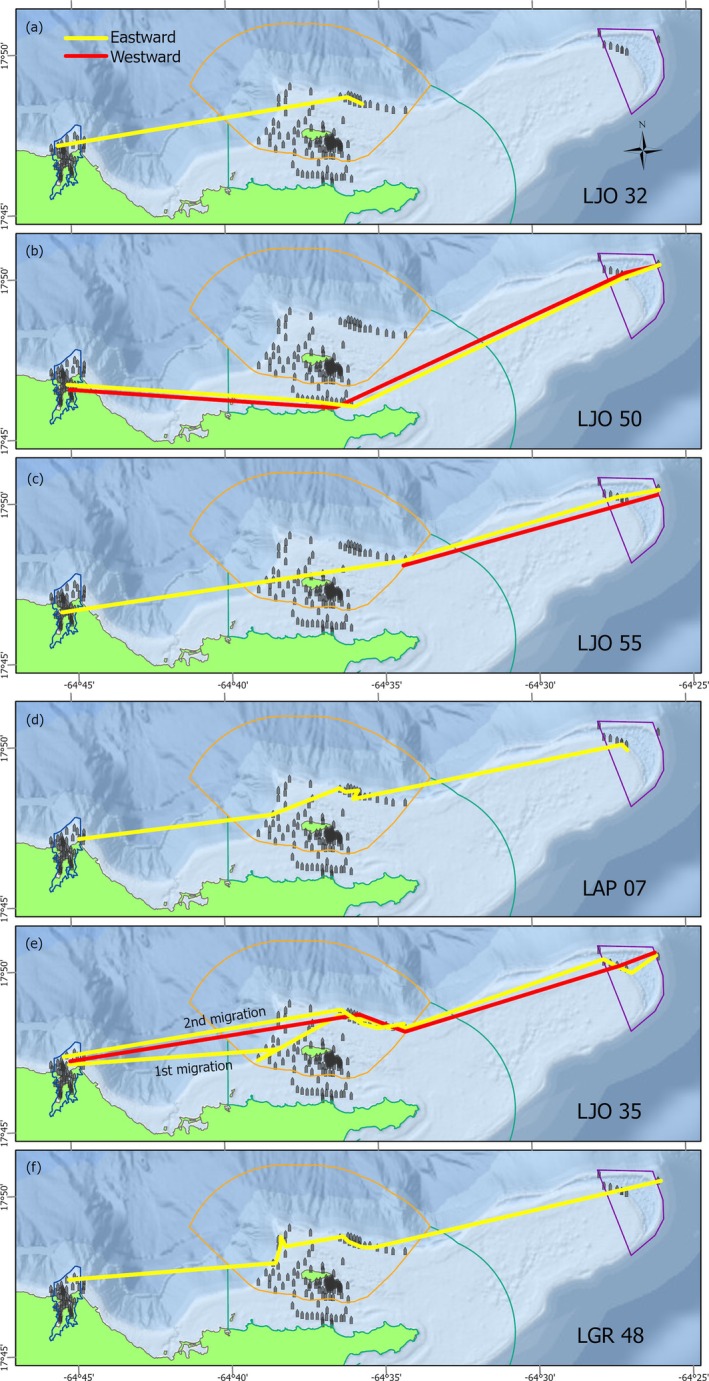
(a–f) Eastward and westward migration tracks for snapper detected on the Buck/Lang array. Movements between receivers are depicted as straight lines between successive detections.

Fifteen snapper (47%) were still detected inside Salt River Bay NHEP when their tags expired 1 year after tagging. These fish were resident to the bay study area < km^2^ in size, and 13 were detected inside the park on >80% of days during that year. The remaining 17 snapper (53%) made possible or confirmed emigrations out of the Salt River array. Six of these fish (four *L. jocu*, one *L. apodus* and one *L. griseus*) were tracked along the Buck/Lang array (Figures 3 and 4). One of these fish (LJO 50; Figure 4b) moved along nearshore reef and hardbottom whereas the others appeared to migrate along the shelf edge reef north of Buck Island. All six made at least one migration past Buck Island (~15 km east of Salt River Bay) and five (Figure 4b–f) travelled all the way to the Lang Bank closure (>30 km east of Salt River Bay), where they were detected by receivers at 32–41 m depth.

These migrations occurred from September to December 2018, with all Lang Bank activity between September 2 and October 17 (later detections were only in Buck Island National Monument). The *L. apodus* and *L. griseus* were both considered mature on migration. One *L. jocu* (LJO 55) was estimated at 29 cm during emigration and still below minimum size of maturity (32 cm; Freitas et al., 2011), but the other three *L. jocu* had grown to an estimated 32–34 cm before emigrating, reaching the minimum threshold but still below *L*_m_ (Table 1).

Average migration speed was 0.36 m s^−1^ (± 0.28 SEM), although most fish had average movement speeds of <0.2 m s^−1^ and only one had a faster speed of 2.9 m s^−1^ occurring over the shortest migration leg analysed (LJO 35; Table 2). It is important to note that these are averages and that detection times on receivers between the start and end points of the migrations suggest that actual movements were not at a continuous and uniform speed. Speed and distance estimates should be considered minimum values due to the straight‐line calculations between receivers. Nine out of 10 migration legs began at night/twilight (Table 2) and the majority of detections during transit (i.e. detections at a given receiver lasting <1 h) were nocturnal, indicating a diel pattern to migrations. There was no apparent relationship between the beginning date of migrations and lunar phase (Table 2), although 0 out of 10 movements began during the new moon. Migrations to Lang Bank took an average of 9.3 days (± 4.1). Most fish had migration transits to Lang Bank of 3–8 days, although one fish took 29 days to reach Lang Bank (LGR 48). For those fish that were detected out at Lang Bank, one was detected for only 2 h before returning westward whereas three others were detected for 2–3 weeks. It is unknown if fish continued into deeper water eastward where the insular shelf drops off rapidly or if they moved elsewhere along the shelf edge promontory out of detection range. Complete migration times including eastward and westward segments as well as time at Lang Bank were 3–4 weeks.

**TABLE 2:** Summary of migration parameters for the six snapper that departed the Salt River array and were detected on the Buck/Lang array, including *Lutjanus apodus* (LAP), *Lutjanus griseus* (LGR) and *Lutjanus jocu* (LJO).

Fish ID	Direction	Departure	Arrival	No. receivers en route	Total distance (km)	Transit time (days)	Mean speed (m s^−1^)	Start time	Lunar quarter at start
LJO 32	Eastward	11‐Nov‐2018	30‐Nov‐2018	3	18	9.0	0.01	Night	Third
LJO 50	Eastward	29‐Sep‐2018	4‐Oct‐2018	4	35.6	3.4	0.14	Day	Third
LJO 50	Westward	17‐Oct‐2018	23‐Oct‐2023	4	35.7	5.3	0.08	Night	Full
LJO 55	Eastward	23‐Aug‐2018	1‐Oct‐2023	9	30.3	8.2	0.04	Night	Full
LJO 55	Westward	1‐Oct‐2023	2‐Oct‐2018	2	15.9	1.3	0.18	Night	Third
LAP 07	Eastward	13‐Sep‐2018	17‐Sep‐2018	8	32.7	4.1	0.09	Night	First
LJO 35	Eastward	28‐Aug‐2018	3‐Sep‐2018	13	37.8	5.3	0.09	Night	Third
LJO 35	Westward	24‐Sep‐2018	25‐Sep‐2018	5	6.3	1.0	2.92	Night	Full
LJO 35	Eastward	10‐Dec‐2018	13‐Dec‐2018	7	19.1	3.3	0.07	Night	First
LGR 48	Eastward	30‐Aug‐2018	28‐Sept‐2018	9	35.1	29.4	0.01	Night	Third

*Note*: For fish that made back and forth movements, there is a row for each leg of their migration.

Two of the six migrating fish returned to the Salt River array (LJO 50 and LJO 35; Figure 4b,e) and appeared to move along similar pathways on both the eastward and westward legs of the migration (e.g. north or south of Buck Island) before returning to the same subarray they previously occupied in the bay (Figure 3). After its first round‐trip migration in September, LJO 35 made a second migration in December along the same receiver pathway and was last detected on Lang Bank (Table 2 and Figure 4e). Three fish were last detected on the northern shelf edge closer to Buck Island, one residing near a receiver there for months (LJO 55; Figures 3 and 4b), suggesting permanent ontogenetic shifts to deeper reef habitat. It should be noted that two of the migrating fish (LJO 35 and LJO 50; Figure 3) were able to relocate between inner Salt River Bay and Buck Island National Monument without reef or canyon receivers capturing their movement, highlighting spatial coverage gaps in the outer Salt River array.

Of the remaining fish that disappeared from the study area but were not detected on the Buck/Lang array, five *L. jocu* had clear departure tracks leaving the Salt River array (e.g. LJO 06, LJO 09, LJO 53; Figure 2). Although they were not detected elsewhere around St Croix, their departures were similar in season and route (i.e. successive detections moving offshore down the canyon) to fish that were tracked migrating east. Estimated total length at the time of emigration suggested that only one was above minimum size at maturity. Thus, the majority of *L. jocu* tracked migrating to the shelf edge and Lang Bank (*n* = 3 of 4) were above minimum maturity size, whereas the majority of *L. jocu* that emigrated but did not appear at the Buck and Lang shelf edge (*n* = 4 of 5) were immature. In total, seven snapper of various species ceased detections before their tag expiration (i.e. ‘possible emigrations’). These lacked an emigration track precipitating their final detection, suggesting they evaded detection as they emigrated or they were removed by predation or fishing (Table 1).

### 3.4 Weekly movements and environmental disturbance response

In the weekly movement simulation and resampling test, only 1 week had detectable movements that were significantly different from a random distribution of fish movements. There were 13 fish movements (i.e. habitat and subarray shifts) observed the week of August 4–10, which never occurred in the 100 random simulations (*p* < 0.01). The number of fish movements in all other weeks of the 2018–2019 tracking span could have arisen by chance if movements were occurring randomly. Most of this activity was from fish in the western bays and marina who shifted that week towards the central bay (e.g. LJO 53; Figure 2), other peripheral bays, the backreef or reef canyon. Of the snapper (*n* = 11) detected regularly on the ‘marina receiver’ (i.e. the innermost receiver) inside the western bay during the 2 weeks prior to 4 August, 10 of those individuals (91%) ceased being detected at that receiver during the week of 4 August, shifting to the mouth of the western bay or outside of it altogether. Some returned to their previous activity space within a month (e.g. LJO 33, LJO 53; Figure 2) whereas others had permanent ontogenetic shifts to other habitats (e.g. LGR 48, LJO 35, LJO 55; Figure 3). There was no relationship between fish size and the nature of movements during this time.

The results of the informal survey of National Park Service and marina staff, in conjunction with National Weather Service and CARICOOS records, yielded three noteworthy anecdotal observations. On 20 September 2017, Hurricane Maria made landfall on St Croix as a Category 5 storm, causing catastrophic damage across the island, damaging or killing mangroves in Salt River Bay and sinking several boats in the marina. Although four receivers were moved, washed onshore or lost, no changes in fish location or movement patterns were detected among any snapper during this period. Second, the Army Corps of Engineers used heavy equipment for a month in December 2017 to dredge and remove sunken boats throughout Salt River Bay. Similarly, no clear changes in fish location or movement were observed during this time.

**FIGURE 5:**
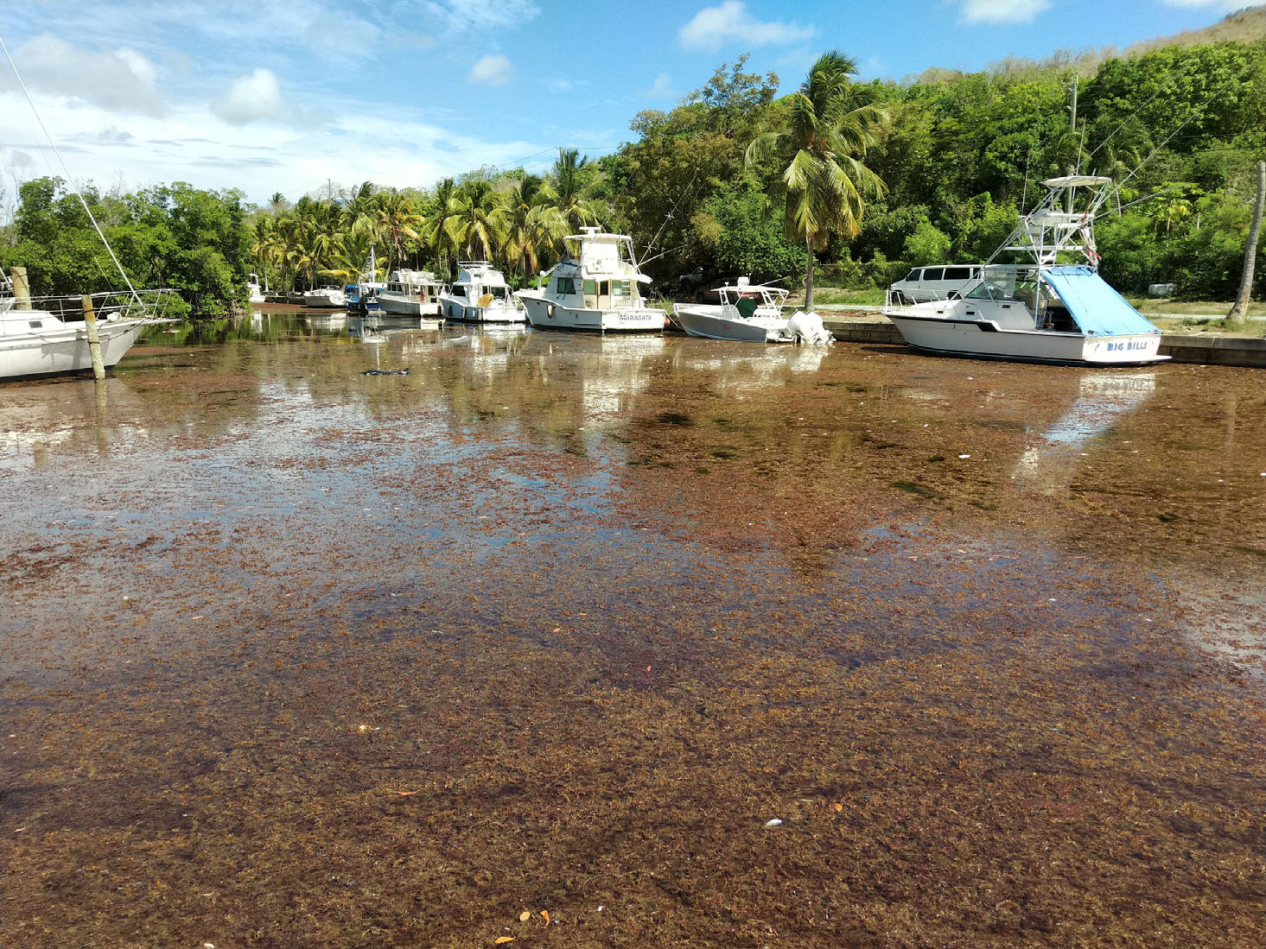
Sargassum influx in Salt River Bay Marina during the week of 4 August 2018. Dead fish can be seen floating on top of sargassum mats (photograph: Richard Peel).

The third notable event, however, occurred during the same week of August 2018 that had significantly more fish movement. During the week of 4 August, managers of the marina reported the most severe incursion of sargassum into the bay that they had witnessed in 17 years of experience in the bay (Peel, R.M. & Peel, G.V., pers. comm.). Of note, respondents were not prompted with any mention of sargassum, only example disturbance events such as marina construction, highlighting the magnitude of this event. During this event, thick mats of floating sargassum entered the bay over the reef crest and through the channel and then were blown to the western side of the bay by the prevailing westward Trade Winds. This floating mass completely covered the surface of marina waters for a few days (Figure 5). As it gradually decomposed and sank, numerous dead fish were observed on the surface throughout the week consisting primarily of baitfish and small (<10 cm) snapper. No other sargassum events of comparable magnitude were observed during the study period and no other environmental disturbances were reported during the study, which corresponded to the week of 4 to 10 August. The CARICOOS Sargassum Response system and Sargassum Watch mapping records (Hu, 2024; Hu et al., 2016) indicate the sargassum wave that occurred in the Caribbean during August 2018 was the most severe such event recorded at that time for the region.

### 3.5 Interactions with management areas

The majority of snapper showed a high degree of site fidelity inside the park until they made permanent emigrations from the area. There was little evidence of fish moving back and forth across the park boundaries aside from the two fish that made round‐trip migrations to Lang Bank and back (Figure 4b,e), and two adult *L. apodus* detected on the five reef receivers <200 m outside the MPA (Figure 1). Ten fish (31% of those tracked) were detected inside the park on a near‐daily basis until their tag expiration, neither emigrating nor showing any sign of crossing the park boundary (e.g. LGR 441, LGR 41, LJO 33; Figure 2). The six fish tracked outside of Salt River Bay NHEP moved across the Buck Island National Monument or EEMP (Figure 4). Three were last detected inside the Monument, with one detected there until tag expiration (LJO 55). Five also travelled on to the Lang Bank closure, but it is important to note that the easternmost receiver on the shelf edge that four fish were detected on (Figure 4) was outside the closure boundary.

## 4 DISCUSSION

Our study is among the first to chronicle incremental shifts of individual snapper to different habitats through ontogeny, from sheltered mangrove creek nurseries to open bays to reefs to the shelf edge habitats where adults may form spawning aggregations. We also sought to address additional data gaps in *L. jocu* ecology, document diel movement patterns in this species and provide the first characterization of an *L. jocu* nursery habitat in the northern hemisphere. The restricted activity areas of the majority of fish in Salt River Bay illustrate the potential conservation benefits of MPAs around mangrove ecosystems, while the documentation of bay to reef ontogenetic shifts and eventual emigrations out of the preserve demonstrate the spillover benefits to declining reef fisheries, both to tropical coastal ecosystems in general and to St Croix in particular. Currently, the only documented snapper spawning aggregation site in St Croix is on the southwest shelf (Heidmann et al., 2024; Kadison et al., 2017). Our data suggest Lang Bank may also be a spawning ground and migratory destination for snappers in addition to other previously documented commercially important species. Finally, this study may be the first to document fine‐scale movement response of a fishery species to periodic sargassum inundation that impacts coastal countries across the Caribbean, Americas and Africa (Louime et al., 2017; Wang et al., 2019).

### 4.1 Diel activity patterns

Some lutjanid species are known to shelter in mangrove structure by day, often as juveniles, or deeper reef structures, often as adults, and move to seagrass at night to forage (Luo et al., 2009; Nagelkerken et al., 2000; Nagelkerken & Velde, 2004; Verweij et al., 2007). Fine‐scale movements documented in this study reinforced those findings: snapper resided by day either among mangroves (mostly immature fish) or on the reef canyon (all mature) and moved from different directions into the central bay and backreef at night. The central bay in the Salt River Bay NHEP contains seagrass and may provide feeding opportunities, while the smaller mangrove‐filled accessory bays as well as the reef and canyon all provide structural refuge (Kendall et al., 2005). Increased detections at night have been attributed to heightened activity while foraging (Hitt et al., 2011; Huijbers et al., 2015). Specifically, *L. jocu* were active on more receivers at night in this study, suggesting they expand their activity area when foraging.

We identified diel patterns in half of the detected fish in this study but there may have been more we could not discern. Inside the bay, small‐scale movements may have been completely contained within the wide detection range of a single receiver. Receivers outside the bay had poorer detection range and spatial gaps in coverage, possibly due to wave action, biotic reef noise and reef obstructions to tag transmissions. Such sporadic detections provide an incomplete picture of movement patterns.

### 4.2 Ontogenetic shifts

*Lutjanus apodus* are known to make an ontogenetic shift from mangroves and seagrass to reefs, including in St Croix (Mateo et al., 2010). The *L. apodus* tagged outside of the bay were adults that likely already completed this transition and the majority resided exclusively on reefs and never foraged inside the bay, similar to populations in other locations (Cocheret de la Morinière et al., 2002; Cocheret de la Morinière, Pollux, Nagelkerken, Hemminga, et al., 2003). *Lutjanus griseus* utilize mangroves and seagrass as juveniles (Faunce & Serafy, 2007) and frequent deeper reefs as adults (Bacheler et al., 2020) but maintain ties to inshore habitats in some locations (Serafy et al., 2003). The two smaller *L. griseus* tagged in mangrove bays made ontogenetic shifts out to the backreef and reefs. The two larger adults (>30 cm) resident on the reef by day likely already made that shift, but still foraged nocturnally inside the bay.

All *L. jocu* in this study were tagged as late juveniles sheltering by day in mangrove habitat, and the majority (*n* = 11, 79%) made stepwise ontogenetic shifts to the open bay and reefs, sometimes emigrating from the study area altogether. *Lutjanus jocu* on the Brazilian coast rely on mangrove‐dominated intertidal creeks and estuaries as juveniles (Monteiro et al., 2009; Pimentel & Joyeux, 2010; Ramos et al., 2011) and visual surveys showing different size classes across habitats indicate ontogenetic shifts from mangrove nurseries to seagrass to progressively deeper reefs offshore as they increase in size (Moura et al., 2011). This may be the first study, however, to observe and document individual *L. jocu* making those incremental movements and behavioural changes from one habitat to another as they mature.

Two *L. jocu* made ontogenetic shifts in the form of dramatic home range expansion to multiple habitats only to reverse course and gradually shift back to their original mangrove bay months later. Ontogenetic shifts may not always be a one‐way process and fishes may make exploratory visits into new habitats before shifting permanently (Kendall, Siceloff, Ruffo, et al., 2021; Kramer & Chapman, 1999; Verweij et al., 2007). Alternatively, this back‐and‐forth movement may reflect periodic fine‐scale shifts in feeding grounds as observed in grunts (Haemulidae) (Appeldoorn et al., 2009) or spawning on the shelf edge, although only one fish was potentially mature at the time.

### 4.3 Migrations and potential spawning activity

Six snapper of various species undertook migrations from the focal study area across coastal shelf and reef habitats, and five were tracked at a known spawning area for other species. The average estimated migration speed of 0.36 m s^−1^ was similar to that reported for lutjanids migrating to spawning habitat elsewhere (Biggs & Nemeth, 2016). Most detections during migrations were nocturnal and we hypothesize that these snapper migrated at night as has been described elsewhere (Huijbers et al., 2015; Luo et al., 2009) and reduced their movement by day, potentially taking shelter at sites out of range of the widely spaced receivers along the coast.

Receivers were deployed on Lang Bank by colleagues because it is a known spawning site for *E. guttatus* and *B. vetula* (Bryan et al., 2019; Nemeth et al., 2006). There are also anecdotal reports of endangered Nassau grouper *Epinephelus striatus* (Bloch 1792) and other groupers spawning on eastern Lang Bank (K. Ewen, pers. comm). However, snapper quickly passed the ridge and basin in the centre of the bank where *E. guttatus* and *B. vetula* spawn and continued on to the eastern shelf edge or likely even past it. Like grouper, lutjanids form spawning aggregations around reef promontories, shelf breaks and other vertical relief (Carter & Perrine, 1994; Claro & Lindeman, 2003; Domeier & Colin, 1997; Heyman et al., 2005), and multiple species of snapper and grouper often spawn at the same site (Heyman & Kjerfve, 2008; Kadison et al., 2006; Lindeman et al., 2000). *Lutjanus jocu* move in and out of the Grammanik Bank aggregation site off St Thomas all year (Biggs & Nemeth, 2016) and make repetitive migrations from other reefs along the shelf edge up to 28 km away (Biggs & Nemeth, unpublished data). Less is known about *L. apodus* and *L. griseus* spawning but *L. griseus* in inshore areas migrate to deeper reefs to spawn (Claro et al., 2009; Domeier & Colin, 1997; Luo et al., 2009) and offshore aggregations are known for *L. griseus* and *L. apodus* in a few locations, often on shelf breaks and reef promontories (Bacheler et al., 2020; Boomhower et al., 2010; Claro & Lindeman, 2003). Based on this knowledge, spawning migrations are the most likely reason for snapper to travel from Salt River Bay to the edge of Lang Bank. The migrations of three *L. jocu* to the shelf edge of Lang Bank during the same 2‐month period, the return migrations of two to their tagging location, and one individual's repetitive migrations to Lang provide strong evidence for *L. jocu* spawning on Lang Bank. A sample size of only one *L. griseus* and one *L. apodus* tracked migrating to Lang with no return migrations is insufficient to conclude that these species spawn on Lang Bank, although their migrations occurred during roughly the same time of year as *L. jocu*. Additional tagging and survey studies during August–December may provide more information about these species' movement patterns and what snapper species spawn on Lang Bank.

Anywhere *L. jocu* were detected on Lang Bank or the northern shelf edge qualifies as suitable spawning habitat based on known aggregation sites (Biggs & Nemeth, 2016; Claro & Lindeman, 2003; Domeier & Colin, 1997; Heyman & Kjerfve, 2008). Most receiver detections on Lang Bank occurred briefly and at night, indicating fish passed them in transit. However, the majority of fish migrated to the easternmost receiver on the shelf edge, which was the only Lang site where fish were detected for over a week and at all times of day, suggesting the eastern tip of Lang Bank was a destination and not a transit point and may be where spawning occurred. More research, including visual surveys and acoustic telemetry, could elucidate the exact location of snapper spawning activity on Lang Bank.

The timing of migrations and detections on Lang Bank suggest that snapper spawning may occur there during September and October. Although snapper may spawn year‐round in at least some areas (Biggs & Nemeth, 2014; França et al., 2021), spawning commonly peaks at certain months and is synchronized to the lunar cycle (e.g. peaking around the full or new moon) (Biggs & Nemeth, 2016; Claro & Lindeman, 2003; Domeier & Colin, 1997; Freitas et al., 2011). *Lutjanus jocu* eggs have even been found to be more viable during new moon and full moon periods (Malanski et al., 2024). We found little correlation between lunar cycle and the timing of migrations or shelf edge activity other than a lack of migrations around the new moon, although the exact dates of spawning activity are unknown. The timing of these individuals' spawning migrations may be influenced by other environmental cues such as changes in temperature, photoperiod or oceanographic currents (Feeley et al., 2018; Heidmann et al., 2024; Heyman et al., 2005).

The size of *L. jocu* that made likely spawning migrations suggest they may reach maturity at a smaller size in St Croix compared to other regions. While the migrating *L. griseus* and *L. apodus* were likely mature given their size (SEDAR, 2022; Stevens et al., 2019), two *L. jocu* were ≤2 cm above minimum size at maturity (Freitas et al., 2011), a third one was 3 cm below it and all were well below the 50 cm estimated *L*_m_ and the average reported adult size of 60 cm (Fröse & Pauly, 2024; Stevens et al., 2019). The tagged *L. jocu* in the current study were smaller than *L. jocu* observed at the Grammanik Bank aggregation off St Thomas (50–76 cm; Biggs & Nemeth, 2016) but comparable to sizes from a Belize aggregation (Carter & Perrine, 1994). Geographic variation in growth and maturity is influenced by different factors such as environmental conditions (Caselle et al., 2011; McMahan et al., 2020) as well as fishing pressure, which can drive decreases in size and age at maturity (Hamilton et al., 2007; Hutchings, 2005; Trippel, 1995). Fishing pressure is a plausible factor here given that lutjanid populations in the US Virgin Islands have declined and average sizes have decreased over several decades, likely due to overexploitation (Beets & Rogers, 2002; Kadison et al., 2017), and *L. jocu* are now rarely encountered in St Croix (Kadison et al., 2017). Life‐history demographics should not be extrapolated across populations and regions if possible (Gray, 2015) and can result in flawed estimates of important management metrics such as reproductive potential and spawning stock biomass. Population‐specific and localized data collection is essential for effective fishery management (Roni & Quinn, 1995; Williams et al., 2016), and the variability in size at maturity among locations highlights the need for length at maturity studies that are specific to US Caribbean populations where data are scarce.

The return migrations of two *L. jocu* back to Salt River Bay's mangroves suggest they may begin spawning before permanently leaving nursery habitats. *Lutjanus jocu* are known to make ontogenetic shifts out of mangrove habitat as they grow, based largely on visual surveys that found individuals to be ≤40 cm in estuarine bays, with larger individuals found only on offshore reefs (Moura et al., 2011). This aligns with our study's results where the largest *L. jocu* caught inside the bay was 39 cm. While some *L. jocu* may linger in nursery habitat after reaching maturity, others may leave before they mature. Several tagged *L. jocu* emigrated from Salt River Bay while still below the estimated minimum size at maturity. These fish may have matured at a smaller size compared to other *L. jocu* populations. Alternatively, ontogenetic changes in dietary and prey needs may drive snapper to new habitats before sexual maturity is reached (Cocheret de la Morinière, Pollux, Nagelkerken, & van der Velde, 2003). The complexity of individual behaviours found in this study further highlights the need for life‐history and ecological studies specific to US Caribbean populations.

Eleven individuals made confirmed or possible emigrations out of the Salt River array to unknown whereabouts and were not detected on the Buck/Lang array afterwards. The ‘possible’ emigrations with no emigration tracks may indicate mortalities, but two snapper migrated between inner Salt River Bay and the Buck/Lang array with no emigration tracks or detections on the reef outside of the bay, demonstrating it was feasible for fish to slip out of the Salt River array undetected. Given that no receivers were located west of Salt River Bay, westward movements into this spatial coverage gap may explain why some of these 11 fish were undetected after leaving the Salt River array. These movements may suggest ontogenetic habitat shifts rather than spawning given that the majority of *L. jocu* in this group were considered immature at the time of emigration. However, if these fish reached maturity at a smaller size than other populations, spawning migrations to the west are also possible and the spawning ground in southwest St Croix is one potential destination. Although it is known as an *L. analis* aggregation site, it is common for multiple snapper species to spawn at the same location (Heyman & Kjerfve, 2008; Lindeman et al., 2000), and cubera snapper *Lutjanus cyanopterus* (G. Cuvier 1828) have been reported at the southwest aggregation site as well (J. Sanchez, pers. comm).

### 4.4 Hurricane and sargassum response

Despite the severe damage inflicted by Category 5 Hurricane Maria in September 2017 to mangroves, boats and infrastructure in Salt River Bay, no snapper relocated during or after the storm. Likewise, neither did sea bream *Archosargus rhomboidalis* (L. 1758) tracked in Salt River Bay during the same period (Kendall, Siceloff, Monaco, et al., 2021) nor *L. analis* tracked in a St Thomas bay (Heidmann et al., 2021). Fishes' behavioural response to storms varies by species (Gutowsky et al., 2021; Munks et al., 2015), but tagging studies have revealed that some fishes change their behaviour or location in response to severe storms. For example, *L. griseus* (Luo et al., 2009), red snapper *Lutjanus campechanus* (Poey 1860) (Patterson III et al., 2001), coastal sharks (Udyawer et al., 2013) and grey triggerfish *Balistes capriscus* Gmelin 1789 (Bacheler et al., 2019) have been observed moving to deeper water offshore as tropical storms and hurricanes approached. We recently tracked *L. griseus* vacating a mangrove creek in south Florida during a hurricane (Kendall et al., in prep.), although that study area was typically <1 m deep and may have afforded less protection from wind and wave action than the 2–5 m deep Salt River Bay and its more extensive submerged mangrove root systems. We conclude that fish responses to even direct hits by severe storms vary not only by species, but also by local physical environment.

Record‐breaking levels of floating sargassum biomass were observed in the Caribbean throughout 2018 (Hu, 2024; Wang et al., 2019). Although there are no quantitative measurements at the granular level of Salt River Bay, anecdotal reports from full‐time residents indicate an unusually severe sargassum event within the study site that resulted in numerous fish deaths. This influx occurred the same week that many snapper exhibited significantly non‐random shifts in location, including every tagged snapper resident to the marina leaving that area, where sargassum buildup was high. Snapper in the marina may have moved to escape the conditions caused by sargassum inundation such as hypoxia and toxic compound accumulation caused by sargassum decomposition (Rodriguez‐Martinez et al., 2019). All movements during this event were conducted by fish in the sheltered bays where sargassum accumulated and remained in place. Fish on the exposed reef habitat outside the bay, where there was no sargassum accumulation, displayed no activity changes. Some snapper that relocated during this sargassum event made permanent habitat shifts. Those inhospitable conditions may have been the root cause of relocation or they may have simply influenced the timing of inevitable ontogenetic shifts.

Most studies on sargassum's impact on coastal fisheries have focused on how influxes physically impede fishers from locating and catching animals and effectively using fishing gear (Cox et al., 2019; Solarin et al., 2014), or how incursions can cause mass mortality events (Rodriguez‐Martinez et al., 2019). Marine species are known to relocate to escape hypoxic zones (Craig & Crowder, 2005; Zhang et al., 2009) and other harmful algal blooms such as red tides (Hallett et al., 2016; Reis‐Filho et al., 2012; Walters et al., 2013). Although fishing communities have reported sargassum to drive some species away from fishing grounds (Ramlogan et al., 2017), we are unaware of any previous studies demonstrating individual fish relocating in response to a sargassum incursion. This study suggests that sargassum influx can alter movement patterns and spur relocation to other areas.

Like storms, fish responses to hypoxia and algal blooms are also species‐specific, as shown by comparing snapper behaviour in this study to that of *A. rhomboidalis*, which were tracked in Salt River Bay during this time (Kendall, Siceloff, Monaco, et al., 2021) but showed no displacement or behavioural change during the sargassum influx event. Furthermore, anecdotal observations suggest that the majority of fish mortalities may have been among snapper and other fish smaller (<10 cm) than fish tagged in this study. The relationship between fish size and hypoxia effects is complex and varied (Müller et al., 2023) but understanding how fishes' size may affect their vulnerability to the negative impacts of sargassum inundation is important to fishery management where this phenomenon occurs. Further research, longer in duration and encompassing more species and size classes, could illuminate long‐term impacts of sargassum inundation to coastal fish communities and the extent to which displacement caused by sargassum is permanent or temporary.

### 4.5 Management implications

Our findings demonstrate Salt River Bay NHEP, one of the few remaining intact mangrove ecosystems in St Croix, provides sheltered mangrove nursery habitat and seagrass feeding grounds for multiple snapper species, and that adjacent reef and canyon habitats support adults as they shift out of the bay. These snapper remain largely within preserve boundaries until making permanent ontogenetic migrations and thus may supply spillover to other areas, demonstrating the MPA's conservation potential. However, no‐take rules and an enforcement strategy for the Salt River Bay NHEP were never finalized or implemented (Gardner, 2002) and fishing is frequently observed in the preserve (pers. obs). Many fish populations on reefs are proportional to the size of adjacent mangrove nurseries (Serafy et al., 2015), and nursery habitat availability outweighs even the impacts of MPAs on reef population numbers (Nagelkerken et al., 2012). Effective protection of these juvenile populations and conservation of their bay habitat is likely to enhance St Croix ecosystems and local fisheries.

The ontogenetic habitat shifts and migrations documented here establish connectivity between MPAs for commercially important species across life stages. However, these areas vary in protection level and all face challenges to effective enforcement. Buck Island National Monument is a no‐take MPA providing reef habitat for adults that have emigrated from Salt River Bay, and a protected migration corridor for fishes travelling east. Receivers were absent between the Salt River and Buck Island arrays, and further study could detail the connectivity between them. Given the available tracking data to the east, however, it is likely the shelf edge between these two managed areas is also a migration corridor that is currently unprotected. EEMP and the Lang Bank closure encompass migration corridors for potential spawners and contain suitable adult shelf edge habitat, but the EEMP's no‐take areas extend <1 km from shore and are unlikely to protect snapper migration.

Our findings support the recognition of Lang Bank as a multispecies spawning ground. Importantly however, the Lang closure boundary stops short of the easternmost receiver that snapper migrated to and their spawning aggregation may occur outside of it. Furthermore, snapper activity in this study occurred on Lang Bank during September–October, earlier than the spawning seasons there for *E. guttatus* (December–February) (Nemeth et al., 2006) and *B. vetula* (November–March) (Bryan et al., 2019), and therefore the existing seasonal no‐take period on Lang Bank from December–March may not offer protection to spawning snapper. More precise information about the eastward extent and timing of snapper spawning relative to the Lang Bank closure is paramount given that fishing on spawning aggregations has caused Caribbean snapper populations and fisheries to decline (Claro et al., 2009; Graham et al., 2008). Enforcement resources, strategy and compliance are also crucial accompaniments to any effective MPA, and poaching has been observed in Buck Island National Monument, EEMP (L. Siceloff and M. Kendall) and the Mutton Snapper Seasonal Closed Area in the southwest (Heidmann et al., 2024). Comprehensive management frameworks address species at all life stages, and a series of connected and enforced MPAs spanning multiple habitats such as these managed areas in St Croix has the potential to protect species throughout their ontogenetic movements.

## AUTHOR CONTRIBUTIONS

M.S.K., C.P., M.E.M. and L.S. conceived of and designed the study and collected data. M.S.K. and M.E.M. obtained funding. L.S. and M.S.K. analyzed and interpreted data and prepared the manuscript.

## Supporting information


**DATA S1** Daily detection plots for additional fish tracked from 2017 to 2018, including *Lutjanus apodus* (LAP), *Lutjanus griseus* (LGR) and *Lutjanus synagris* (LSY).

**DATA S2** Daily detection plots for additional fish tracked from 2018 to 2019, including *Lutjanus apodus* (LAP) and *Lutjanus synagris* (LSY).

**DATA S3** Daily detection plots for additional fish tracked from 2018 to 2019, including *Lutjanus analis* (LAN), *Lutjanus griseus* (LGR) and *Lutjanus jocu* (LJO).
