# Supplementary figures and images for: Wayward youth: how maturity, reproduction and seaweed drive snapper (Lutjanus spp.) habitat shifts
Source: J Fish Biol. 2025 Sep 8;107(6):2106–24. doi: 10.1111/jfb.70212 (PMC12861840; doi:10.1111/jfb.70212)

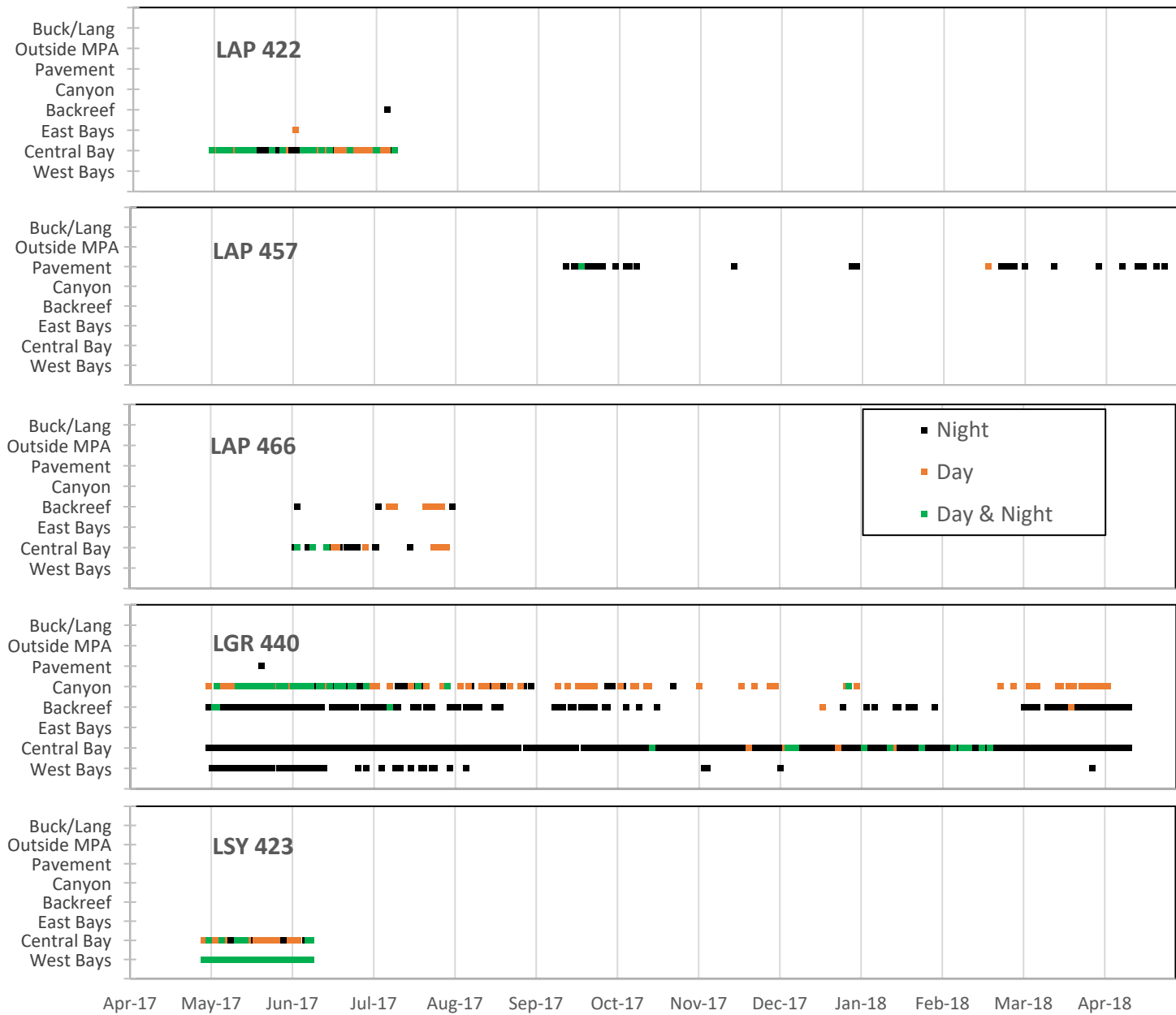

Supplement: Supplementary file 1 — DATA S1 Daily detection plots for additional fish tracked from 2017 to 2018, including Lutjanus apodus (LAP), Lutjanus griseus (LGR) and Lutjanus synagris (LSY). [file JFB-107-2106-s002.pdf]

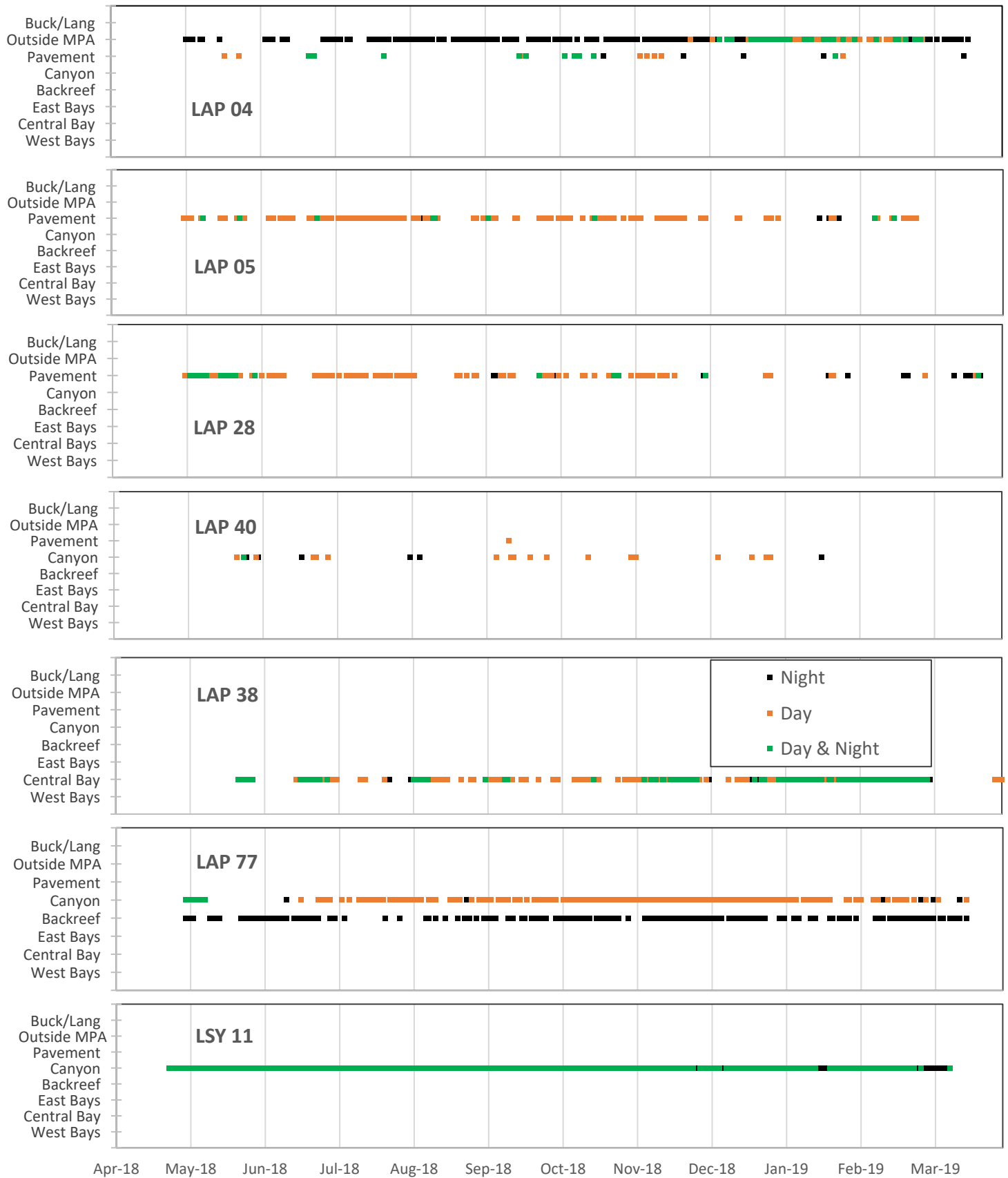

Supplement: Supplementary file 2 — DATA S2 Daily detection plots for additional fish tracked from 2018 to 2019, including Lutjanus apodus (LAP) and Lutjanus synagris (LSY). [file JFB-107-2106-s001.pdf]

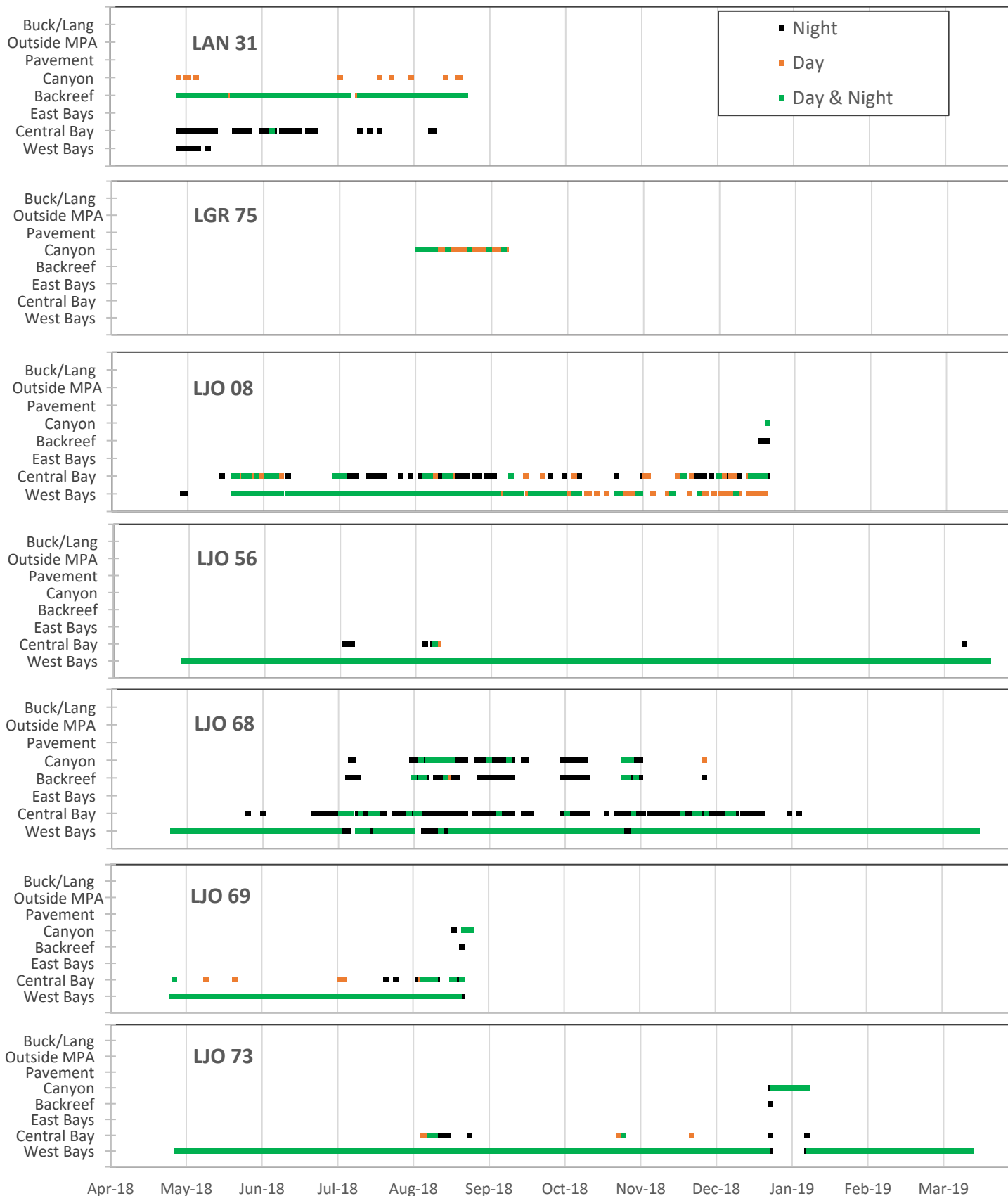

Supplement: Supplementary file 3 — DATA S3 Daily detection plots for additional fish tracked from 2018 to 2019, including Lutjanus analis (LAN), Lutjanus griseus (LGR) and Lutjanus jocu (LJO). [file JFB-107-2106-s003.pdf]
